# The Cardiac Ryanodine Receptor Provides a Suitable Pathway for the Rapid Transport of Zinc (Zn^2+^)

**DOI:** 10.3390/cells11050868

**Published:** 2022-03-03

**Authors:** Jana Gaburjakova, Marta Gaburjakova

**Affiliations:** Centre of Biosciences, Institute of Molecular Physiology and Genetics, Slovak Academy of Sciences, Dubravska cesta 9, 840 05 Bratislava, Slovakia

**Keywords:** cardiac muscle, ryanodine receptor, zinc (Zn^2+^), conductance, permeability coefficient, planar lipid membrane

## Abstract

The sarcoplasmic reticulum (SR) in cardiac muscle is suggested to act as a dynamic storage for Zn^2+^ release and reuptake, albeit it is primarily implicated in the Ca^2+^ signaling required for the cardiac cycle. A large Ca^2+^ release from the SR is mediated by the cardiac ryanodine receptor (RYR2), and while this has a prominent conductance for Ca^2+^ in vivo, it also conducts other divalent cations in vitro. Since Zn^2+^ and permeant Mg^2+^ have similar physical properties, we tested if the RYR2 channel also conducts Zn^2+^. Using the method of planar lipid membranes, we evidenced that the RYR2 channel is permeable to Zn^2+^ with a considerable conductance of 81.1 ± 2.4 pS, which was significantly lower than the values for Ca^2+^ (127.5 ± 1.8 pS) and Mg^2+^ (95.3 ± 1.4 pS), obtained under the same asymmetric conditions. Despite similar physical properties, the intrinsic Zn^2+^ permeability (P_Ca_/P_Zn_ = 2.65 ± 0.19) was found to be ~2.3-fold lower than that of Mg^2+^ (P_Ca_/P_Mg_ = 1.146 ± 0.071). Further, we assessed whether the channel itself could be a direct target of the Zn^2+^ current, having the Zn^2+^ finger extended into the cytosolic vestibular portion of the permeation pathway. We attempted to displace Zn^2+^ from the RYR2 Zn^2+^ finger to induce its structural defects, which are associated with RYR2 dysfunction. Zn^2+^ chelators were added to the channel cytosolic side or strongly competing cadmium cations (Cd^2+^) were allowed to permeate the RYR2 channel. Only the Cd^2+^ current was able to cause the decay of channel activity, presumably as a result of Zn^2+^ to Cd^2+^ replacement. Our findings suggest that the RYR2 channel can provide a suitable pathway for rapid Zn^2+^ escape from the cardiac SR; thus, the channel may play a role in local and/or global Zn^2+^ signaling in cardiomyocytes.

## 1. Introduction

Zinc (Zn^2+^) is one of the most abundant metal cations in mammalian cells, with diverse functions in numerous physiological processes important for differentiation, growth and survival (reviewed in [1,2]). Although, Zn^2+^ has been generally considered to have moderate biological importance, significant advances in understanding Zn^2+^ biology in the past decade have changed this view. Reflecting the physiological relevance, both Zn^2+^ deficiency and excess have been documented in a wide range of pathological conditions, including cancer, diabetes and inflammatory, cardiovascular and neurodegenerative diseases, which have been the topic of several review articles [3,4,5,6,7,8,9]. While catalytic and structural functions of Zn^2+^ are well-established in a great number of metalloproteins [10,11], Zn^2+^ signaling capacities have only recently received extensive attention, mainly in a cellular and molecular context.

The Zn^2+^ affinity to protein metal binding sites is highly competitive towards Mg^2+^ and Ca^2+^, therefore, free intracellular Zn^2+^ concentration is tightly controlled and fluctuates in an extraordinarily narrow range (pM–nM), depending on the cell type [12,13,14,15]. In addition to cytosolic Zn^2+^-chelating proteins (reviewed in [16]), various Zn^2+^ transporters (carrier-type) are involved in controlling intracellular Zn^2+^, such as ZnT proteins exporting Zn^2+^ from the cytoplasm and ZIP proteins with the opposite function (reviewed in [17,18,19]). 

ZnT and ZIP proteins have a distinct subcellular location, depending on their contribution to Zn^2+^ mobilization. In mammals, they are mostly localized to the plasma membrane [20,21,22,23,24,25,26] or in membranes of intracellular organelles, such as the ER and Golgi apparatus [27,28,29,30,31,32,33]. All these intracellular compartments obviously accumulate Zn^2+^ [34,35,36], but only some could be implicated in Zn^2+^ signaling, exemplified by Zn^2+^ transients and waves [35,37]. Although, there is no clear evidence, it can be proposed that the SR in cardiomyocytes acts as a dynamic storage for Zn^2+^ cations, readily available for release [8,38]. These results strongly imply the presence of Zn^2+^ transporters, and indeed ZIP and ZnT proteins have been evidenced in the heart tissue [27,39,40]. Moreover, their predominant subcellular localization in the SR membrane has been demonstrated [41].

It is well established that the release and accumulation of Ca^2+^ by the SR in cardiomyocytes is fundamental to the excitation-contraction coupling [42,43,44]. The cyclic release of Ca^2+^ required for contraction is mediated by the cardiac ryanodine receptor (RYR2) displaying a remarkable conductance for Ca^2+^ [45,46] compared to other Ca^2+^-permeable channels [47,48]. However, the RYR2 channel discriminates only slightly between divalent cations [46], and thus has been shown to be also permeable to Mg^2+^, Sr^2+^ and Ba^2+^ [46,49,50,51]. Since we noticed that both Mg^2+^ and Zn^2+^ possess similar physical properties relevant to permeation through ion channels [52,53,54], we examined whether the RYR2 channel provides a suitable permeation pathway for Zn^2+^. At the single-channel level, we established that Zn^2+^ greatly permeated the RYR2 channel in the lumen-to-cytosol direction, although with a lower conductance and intrinsic permeability than Ca^2+^ and even Mg^2+^. Our results indicate that the RYR2 channel may play a role in Zn^2+^ signaling in cardiomyocytes, and the channel itself could be a direct target for the localized Zn^2+^ increase, with the Zn^2+^ finger, a well-known Zn^2+^-binding site, extending into the cytosolic vestibular portion of the RYR2 permeation pathway [55].

## 2. Materials and Methods

### 2.1. Single-Channel Recordings

The sarcoplasmic reticulum (SR) microsomes enriched in RYR2 channels isolated from rat ventricular muscle were used for single-channel recordings as described previously [51] with some modifications. Planar lipid membranes (BLMs) were formed across a 50–70 μm aperture in the wall of a polystyrene cup separating two compartments, cytosolic and luminal. 

The cytosolic compartment was filled with 1 mL of 50 mM KCl, 10 mM Tris and 20 mM HEPES (pH = 7.35). The free cytosolic Ca^2+^ concentration of 90 nM was obtained by including 1 mM ethylene glycol-bis(β-aminoethylether)–*N*,*N*,*N*′,*N*′-tetraacetic acid (EGTA) and 0.587 mM CaCl_2_. In some cases, *N*,*N*,*N*′,*N*′-tetrakis(2-pyridylmethyl)-ethylene diamine (TPEN) or nitrilotriacetic acid (NTA) was used to chelate Zn^2+^ impurities in the cytosolic solutions. The free Zn^2+^ ([Zn^2+^]_C_) and Ca^2+^([Ca^2+^]_C_) concentrations were determined by WinMaxc32 version 2.50 (http://www.stanford.edu/~cpatton/maxc.html, accessed on 24 April 2021). In control experiments, the luminal compartment was filled with 1 mL of 1–8 mM Ca(OH)_2_, 50 mM KCl, 10 mM Tris and 22–43 mM HEPES (pH = 7.35). In mole-fraction experiments for luminal Mg^2+^ or Ca^2+^, prior to formation of the BLM, 4–7 mM of MgCl_2_ or CaCl_2_ was added to the luminal solution to obtain varied mixtures with 1–4 mM Ca(OH)_2_ while maintaining the total divalent concentration at 8 mM. For pure Mg^2+^ or Ca^2+^, the luminal compartment was filled with 1 mL of 8 mM MgCl_2_ or CaCl_2_, 50 mM KCl, 10 mM Tris and 20 mM HEPES (pH = 7.35). The fusion of SR microsomes with the BLM was not successful enough when Zn^2+^ was present on the BLM luminal side, therefore, the RYR2 channel was exposed to 8 mM ZnCl_2_, 50 mM KCl, 10 mM Tris and 20 mM HEPES (pH = 6.83) by perfusing the luminal compartment after channel reconstitution under control conditions. In mole-fraction experiments, 4–7 mM ZnCl_2_ was also added to 1–4 mM Ca(OH)_2_ after RYR2 reconstitution. In addition, mixtures of 8 mM Ca(OH)_2_ with 8 mM ZnCl_2_ or 8 mM CdCl_2_ were also tested. In the control and for the MCl_2_ additions (4–7 mM), 1–2 mM cytosolic caffeine was used to moderately activate RYR2 channels (M: Mg, Zn or Ca). Similarly, these caffeine concentrations were sufficient for the activation of channels exposed to 8 mM Ca(OH)_2_ mixed with 8 mM ZnCl_2_ or 8 mM CdCl_2_. When ZnCl_2_ or MgCl_2_ was present in the luminal solution alone, caffeine concentration was increased to 6–7 mM to achieve a moderate RYR2 activity. The channel was incorporated into the BLM in a fixed orientation with the cytosolic side always exposed to the cytosolic solution because all RYR2 channels were sensitive to cytosolic caffeine [55,56,57,58]. At the end of experiments, the addition of ryanodine causing the characteristic transition to a half-conducting state was used to validate the channel identity [59].

### 2.2. Acquisition and Analysis of Single-Channel Recordings

Data acquisition and analysis of the open probability (P_O_), frequency of opening (F), average open (T_O_) and closed times (T_C_) were performed as detailed in [51,60]. The conductance (G) and the reversal potential (E_rev_) were determined from a linear regression of the current-voltage relationship, acquired by applying membrane voltage varying from −20 mV to +20 mV. The slope of the fitted line was equal to G, and E_rev_ was taken as the intersection of the voltage axis with the linear fit. To avoid misinterpretation of E_rev_ measurements, the E_rev_ value was further corrected for the measured offset voltage at the end of the experiment and liquid junction potentials (LJPs). The LJP values were determined for all tested luminal solutions according to Barry et al. [61] and were in the range from −0.18 mV to +0.76 mV. The current amplitude at 0 mV (I_0_) was consequently corrected. For tested M^2+^ (M^2+^: Mg^2+^, Zn^2+^ or Ca^2+^), the dependence of E_rev_ on a composition of M^2+^/Ca^2+^ mixture on the RYR2 luminal face was fitted by the following equation to determine the relative Ca^2+^/M^2+^ permeability coefficient (P_Ca_/P_M_), referred to as intrinsic permeability [62]:

(1) $$E_{\mathrm{rev}}=\frac{\mathrm{RT}}{2F}\;\ln\frac{4P_{\mathrm{Ca}}[{\mathrm{Ca}}^{2+}{]}_{C}\;+{\;P}_{K}[K^{+}{]}_{C}\;+\;P_{\mathrm{Tris}}[{\mathrm{Tris}}^{+}{]}_{C}}{4P_{\mathrm{Ca}}[{\mathrm{Ca}}^{2+}{]}_{L}\;+\;P_{K}[K^{+}{]}_{L}\;+\;P_{\mathrm{Tris}}[{\mathrm{Tris}}^{+}{]}_{L}\;+\;4P_{M}[M^{2+}{]}_{L}},$$

Equation (1) is derived from the well-known Goldman—Hodgkin—Katz (GHK) equation [63,64]; where F is Faraday’s constant, R is the universal gas constant and T is the absolute temperature. At room temperature (25 °C), RT/F is ~25.693 mV. The values of P_Ca_/P_Tris_ and P_Ca_/P_K_ were, respectively, 29.545 [65] and 6.5 [46,66]. The subscripts C and L denote a cytosolic or luminal quantity, respectively. Similarly, the E_rev_ plotted against pure luminal Ca^2+^ ([Ca^2+^]_L_) was fitted. For visualization purposes, the dependences of G and I_0_ on [Ca^2+^]_L_ were fitted by the Michaelis—Menten equation. 

Ion diffusion theory [67] was used to relate the local [Zn^2+^], Zn^2+^ current amplitude at 0 mV (I_0,Zn_), and distance from an open channel pore (r) (Equation (2)):

(2) $$\left[{\mathrm{Zn}}^{2+}\right]\left(r\right)=e^{\frac{-r}{\sqrt{\frac{D_{\mathrm{Zn}}}{k\left[B\right]}}}}\frac{I_{0,\mathrm{Zn}}}{4{\pi \mathrm{FD}}_{\mathrm{Zn}}r}.$$

Here, D_Zn_ = 7.03 × 10^−10^ m^2^ s^−1^ [68] is the diffusion coefficient for Zn^2+^, k is the rate for Zn^2+^ binding to the buffer B, [B] = 1 mM is the buffer concentration, F = 96,500 C mol^−1^ is the Faraday constant. The only Zn^2+^ buffer present in most of our experiments was EGTA and its rate of Zn^2+^ binding, k, was 2.6 × 10^6^ M^−1^ s^−1^ [69].

The results are reported as the average ± SEM or the fitted value ± SEM. Statistical comparison of differences in mole-fraction experiments (I_0_ and G) were made by two-way ANOVA with Tukey’s post hoc test. We also used one-way ANOVA with Tukey’s post hoc test to statistically compare changes in the pH of solutions when luminal M^2+^ was added and changes in the values of T_O_, T_C_, and F caused by luminal Zn^2+^. Unpaired Student’s *t* tests were performed to detect significant changes in the latency of RYR2 activity decay when [Cd^2+^]_L_ was added and differences in I_0_, E_rev_, or G values when pH was changed. Paired Student’s *t* tests were utilized for statistical comparison of differences in I_0_, E_rev_, or G values when 8 mM [Zn^2+^]_L_ or 8 mM [Cd^2+^]_L_ was mixed with 8 mM [Ca^2+^]_L_, and changes in P_O_ when NTA or TPEN was added to chelate Zn^2+^ in the cytosolic solution. Differences were regarded to be statistically significant at *p* < 0.05.

## 3. Results

RYR2 channels isolated from rat hearts were incorporated into the BLMs and recorded under voltage clamp conditions. The goal here was to measure the RYR2 current carried by Zn^2+^ in the lumen-to-cytosol direction. Data are presented that define permeation and gating properties including the intrinsic Zn^2+^ permeability of the RYR2 channel. Additionally, a potential role of the RYR2 Zn^2+^ current in local Zn^2+^ signaling was assessed by computing Zn^2+^ accumulation near potential molecular targets, such as neighboring RYR2 channels and the Zn^2+^ finger domain extending into the cytosolic vestibular portion of the RYR2 permeation pathway [55]. Finally, the ability of Cd^2+^ current in the lumen-to-cytosol direction to displace Zn^2+^ from the RYR2 Zn^2+^ finger was investigated.

Working with Zn^2+^ compounds is usually difficult, because they show a low solubility in water at the physiologically-relevant pH range [70,71]. We therefore tested more Zn^2+^ compounds and found that the chloride salt of Zn^2+^ was most appropriate for our purpose. In addition, 8 mM ZnCl_2_ was the maximal concentration available for testing to avoid formation of precipitates and thus prevent overestimation of the Zn^2+^ content. Of note, K^+^ and Tris^+^ cations were also added to the solutions, although both permeate the RYR2 channel [65] and could potentially interfere with RYR2 permeation properties for Zn^2+^ cations. However, they were added symmetrically to both RYR2 sides to diminish their contributions. Since only the chloride salt of Zn^2+^ could have been used, a small Cl^−^ gradient (50 mM vs. 58–66 mM) was inevitably generated at 0 mV by Zn^2+^ additions to the luminal solution, thus providing a driving force for Cl^−^ transport through the BLM. Despite the presence of Cl^−^ channels in cardiac SR microsomes, we did not observe their activity under the conditions utilized in our study. The reason for this probably lies in the fact that Cl^−^ gradient was not sufficient to produce recordable Cl^−^ currents. Another possibility is that Cl^−^ channels were not incorporated into the BLMs together with RYR2 channels.

### 3.1. The RYR2 Conductance for Zn^2+^

**Figure 1:**
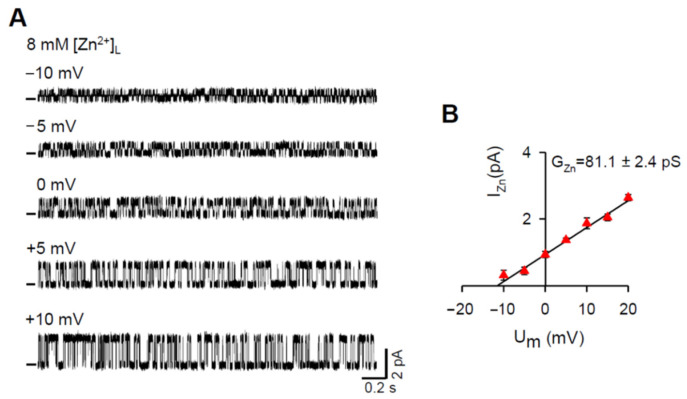
The RYR2 channel is permeable to Zn^2+^. (**A**) Representative RYR2 currents (digitally filtered at 250–400 Hz), shown as upward deflections from the marked zero-current level, in the presence of 8 mM [Zn^2+^]_L_ when Zn^2+^ was only the charge carrier. Current recordings were conducted at −10, −5, 0, +5, +10 mV and are from the same channel. RYR2 channels were activated by 6–7 mM caffeine in the presence of 90 nM free [Ca^2+^]_C_. (**B**) Current-voltage relationship of the RYR2 channel was well-fitted by a straight line with the G_Zn_ value of 81.1 ± 2.4 pS. Data shown are average ± SEM of 5–7 experiments and error bars are shown only when SEM is larger than symbol size.

Our initial aim was to directly record the Zn^2+^ current by building the Zn^2+^ gradient across the BLM. However, after several unsuccessful attempts to reconstitute RYR2 channels with Zn^2+^ in the luminal solution, we modified our experimental approach. The luminal compartment was perfused with the Zn^2+^ solution after RYR2 channels were incorporated into the BLMs in the presence of 8 mM [Ca^2+^]_L_. Representative RYR2 recordings measured in the presence of 8 mM [Zn^2+^]_L_ are shown in Figure 1A. The channels were solely activated by 6–7 mM cytosolic caffeine in the presence of non-activating free [Ca^2+^]_C_ (90 nM) to record the longer and fully resolved open and closed channel events required for the unbiased determination of the Zn^2+^ current values (I_Zn_). When the membrane voltage changed in 5 mV incremental steps from −10 mV to +10 mV, the RYR2 I_Zn_ increased from 0.31 ± 0.15 pA to 1.86 ± 0.16 pA. At 0 mV, the typical value of I_0,Zn_ was 0.927 ± 0.093 pA. To estimate Zn^2+^ conductance (G_Zn_), linear regression was performed on the current-voltage relationship (Figure 1B). Under asymmetrical conditions when the pure Zn^2+^ current was driven by the 8 mM gradient, the value of G_Zn_ was 81.1 ± 2.4 pS. Moreover, the reversal potential (E_rev_) was −11.8 ± 1.1 mV.

### 3.2. Mole-Fraction Experiments under Non-Saturating Conditions

**Figure 2:**
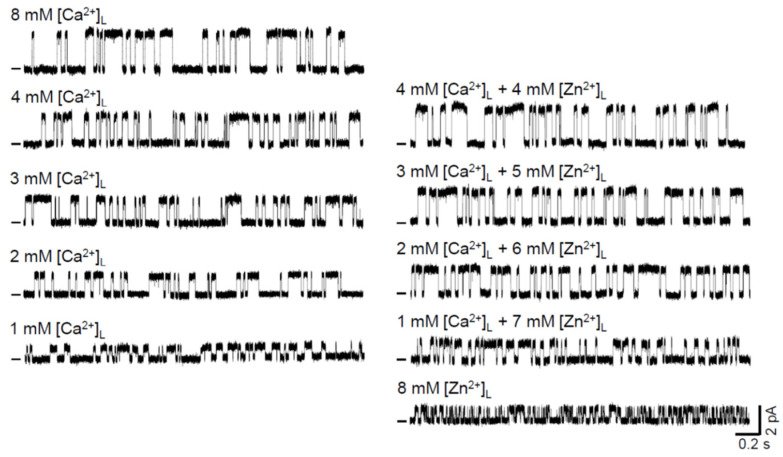
Luminal Zn^2+^ mixed with Ca^2+^ increases RYR2 currents under non-saturating conditions. Representative RYR2 currents (digitally filtered at 250–400 Hz), shown as upward deflections from the marked zero-current level, in the absence (control, left five traces) and after luminal Zn^2+^ exposure (right five traces). In total, 4, 5, 6, 7 mM [Zn^2+^]_L_ was added to 4, 3, 2, 1 mM [Ca^2+^]_L_ (control), respectively, to keep the total divalent concentration constant at 8 mM. Representative RYR2 recordings for pure 8 mM [Ca^2+^]_L_ (control) and 8 mM [Zn^2+^]_L_ are also shown. RYR2 channels were activated by 1–2 mM caffeine in the presence of 90 nM free [Ca^2+^]_C_. In the presence of 8 mM [Zn^2+^]_L_, caffeine concentration was increased to 6–7 mM. Current recordings were conducted at 0 mV and are from different channels.

To evaluate the Zn^2+^ permeability coefficient relative to Ca^2+^, we investigated the mole-fraction behavior of I_0_, E_rev_, and G in the following Zn^2+^/Ca^2+^ mixtures on RYR2 luminal face. Particularly, 4, 5, 6 and 7 mM [Zn^2+^]_L_ was mixed with 4, 3, 2 and 1 mM [Ca^2+^]_L_, respectively. We focused on the luminal solutions with the highest mole fraction of Zn^2+^ because if there were differences from the control (1–8 mM [Ca^2+^]_L_), they should be the most pronounced. Figure 2 depicts the representative RYR2 recordings at 0 mV in the control and when luminal Zn^2+^ was present. RYR2 channels were moderately activated by 1–2 mM caffeine and only for 8 mM [Zn^2+^]_L_, caffeine concentration was increased to 6–7 mM because luminal Ca^2+^, in contrast with other divalent cations, has been shown to be a strong sensitizer of the RYR2 channel to caffeine [50,51]. Since RYR2 gating is also strongly affected by luminal Ca^2+^ [51], the RYR2 channel exhibited a much faster gating, manifested by the increased number of open and closed events at the similar channel activity when only 8 mM [Zn^2+^]_L_ was present (Figure 2). At the quantitative level, this finding was supported by gating parameter calculations. Results collected for the frequency of opening (F), average open (T_O_) and closed (T_C_) times are summarized in Table 1. The F value was increased more than three-fold when 8 mM [Ca^2+^]_L_ was completely replaced by 8 mM [Zn^2+^]_L_. Because F is the reciprocal of a summation of T_O_ and T_C_, a significant shortening in both T_O_ and T_C_ was observed. As we expected, the presence of even 1 mM [Ca^2+^]_L_ precluded any change in RYR2 gating behavior when luminal Zn^2+^ was added. This is fully consistent with findings gained for various mixtures of luminal Mg^2+^ or Ba^2+^ with Ca^2+^ [51], thus further highlighting a specific ability of luminal Ca^2+^ to slow down RYR2 gating. Moreover, no appreciable differences were noted in all three gating parameters calculated for 8 mM [Zn^2+^]_L_ and 8 mM [Mg^2+^]_L_, similar to that found previously for luminal Mg^2+^ and Ba^2+^ [51]. 

**Figure 3:**
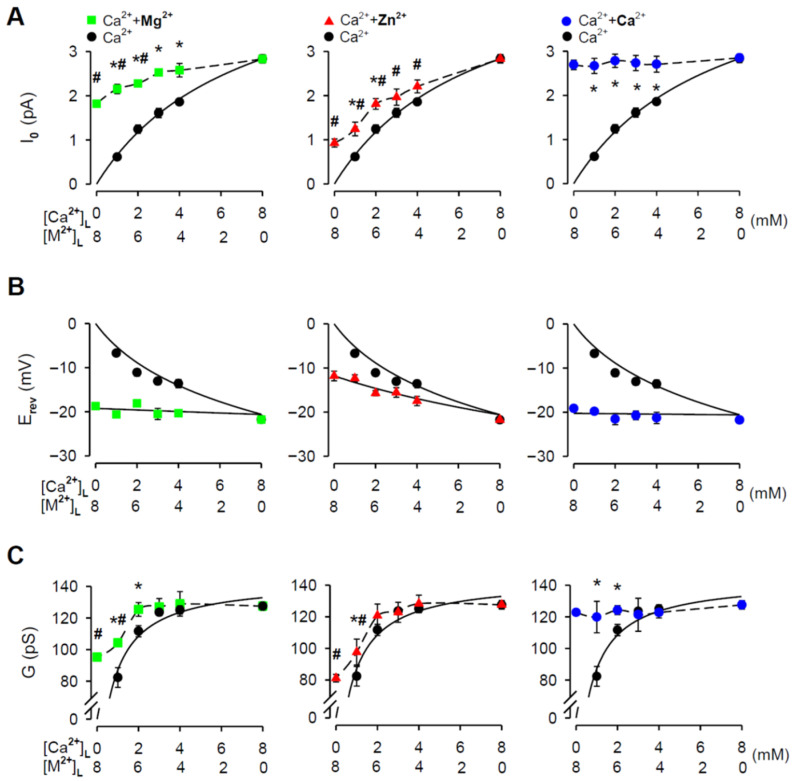
M^2+^/Ca^2+^ mole-fraction experiments under non-saturating conditions. The I_0_ (**A**), E_rev_ (**B**), and G (**C**) plotted against a composition of M^2+^/Ca^2+^ mixture on the RYR2 luminal face (M^2+^: Mg^2+^–green square, Zn^2+^–red triangle, or Ca^2+^–blue circle). In total, 4, 5, 6, 7 mM [M^2+^]_L_ was added to 4, 3, 2, 1 mM [Ca^2+^]_L_, respectively, to keep the total divalent concentration constant at 8 mM. Data obtained for pure 8 mM [M^2+^]_L_ are also included. As the control, the [Ca^2+^]_L_-dependence of E_rev_, G and I_0_ is shown (black circle). * Significantly different from the respective control values; ^#^ significantly different from the control value obtained for 8 mM [Ca^2+^]_L_ (two-way ANOVA with Tukey’s post hoc test). The solid lines in (**B**) are the best fits using Equation (1) and in (**A**) and (**C**) using the Michaelis—Menten equation. The dashed lines in (**A**) and (**C**) are drawn point to point. Data shown are average ± SEM of 5–15 experiments and error bars are shown only when SEM is larger than symbol size.

Further, it is evident from the raw current traces that for all tested Zn^2+^ additions the I_0_ substantially increased (Figure 2). The averaged results are presented in Figure 3A (middle panel). In the control, the I_0_ grew with increasing [Ca^2+^]_L_. The dependence of I_0_ on [Ca^2+^]_L_ was well fitted by the Michaelis—Menten equation. The significant increase in I_0_ by ~45% and ~100% was caused by the addition of 6 mM and 7 mM [Zn^2+^]_L_, respectively. For lower [Zn^2+^]_L_, an increasing trend was also detected; however, it did not achieve significance. To demonstrate the validity and reliability of our Zn^2+^ measurements, we generated the same dataset for Mg^2+^ and Ca^2+^ (chloride salts). Mg^2+^ and Zn^2+^ share the intrinsic physical properties such as ionic size, charge density and hydration enthalpy (Table 2) relevant to permeation through ion channels [52,53,54]; therefore, we would expect similar passage of these cations through the RYR2 pore. As the positive control, varied CaCl_2_ concentrations were added to the luminal RYR2 side to reach the total 8 mM [Ca^2+^]_L_. Here, M^2+^ stands for Mg^2+^, Zn^2+^ or Ca^2+^ for simplicity. Luminal Zn^2+^ had less ability to affect I_0_ carried by Ca^2+^ than luminal Mg^2+^. This was particularly noted when 7 mM [Mg^2+^]_L_ caused more than three-fold increase in the RYR2 current driven by the 1 mM Ca^2+^ gradient (Figure 3A, left panel). Lower [Mg^2+^]_L_ had a smaller but still significant impact. Because the RYR2 I_0_ was considerably lower when 8 mM [Ca^2+^]_L_ was replaced by 8 mM [Mg^2+^]_L_, the dominance of Mg^2+^ (6–7 mM) in the mixture with Ca^2+^ was not sufficient to reach the value of I_0_ with pure 8 mM [Ca^2+^]_L_. Therefore, only the Ca^2+^ additions (chloride salt) caused such a large increase that the I_0_ values became similar to those obtained for both 8 mM Ca(OH)_2_ (2.835 ± 0.094 pA) and 8 mM CaCl_2_ (2.69 ± 0.11 pA) (Figure 3A, right panel). I_0_ data indicate that the RYR2 channel apparently has a lower conductance and intrinsic permeability for Zn^2+^ than for Ca^2+^ and Mg^2+^. 

We tested this possibility by determining E_rev_ and G from current-voltage relationships when the membrane voltage changed in 5 mV incremental steps from −20 mV to +20 mV. All collected current-voltage plots were Ohmic (data not shown) and well fitted by a straight line. In the control, the E_rev_ values substantially decreased with raising [Ca^2+^]_L_ from 1 mM to 8 mM (Figure 3B). Equation (1) well reproduces the luminal Ca^2+^-dependence of E_rev_, thus validating the accuracy of our E_rev_ determination. When luminal Zn^2+^ was added, the E_rev_ decreased, as expected for the addition of permeant cation, while E_rev_ for pure 8 mM [Zn^2+^]_L_ was −11.8 ± 1.1 mV (Figure 3B, middle panel). To estimate the intrinsic Zn^2+^ permeability of the RYR2 channel, the dependence of E_rev_ on a composition of Zn^2+^/Ca^2+^ mixture was then fitted by Equation (1) yielding the relative Ca^2+^/Zn^2+^ permeability coefficient of 2.65 ± 0.19 (P_Ca_/P_Zn_). In comparison, the E_rev_ data collected for Mg^2+^/Ca^2+^ mixtures were well fitted with Equation (1) when P_Ca_/P_Mg_ was 1.146 ± 0.071 (Figure 3B, left panel). This is in good agreement with the intrinsic Mg^2+^ permeability reported for the RYR2 channel [46]. As expected, the E_rev_ measured in Ca^2+^/Ca^2+^ mixtures did not depend on a mixture composition because 8 mM [Ca^2+^]_L_ was always present on the RYR2 luminal face. The fitted value for P_Ca_/P_Ca_ was equal to 1.035 ± 0.061 (Figure 3B, right panel). 

In general, ion translocation through the channel pore is characterized by a specific conductance, therefore, we examined G as a function of the Zn^2+^/Ca^2+^ mixture composition (Figure 3C, middle panel). For the pure luminal Ca^2+^ solutions, the G values plotted against [Ca^2+^]_L_ were fitted by the Michaelis—Menten equation with almost full saturation at 8 mM [Ca^2+^]_L_ (Figure 3C). When luminal Zn^2+^ was added, we revealed a significant increase in G only for the addition of 7 mM [Zn^2+^]_L_ to 1 mM [Ca^2+^]_L_ (Figure 3C, middle panel), evidently because of the weaker intrinsic Zn^2+^ permeability (P_Ca_/P_Zn_ = 2.65 ± 0.19) and a significantly lower RYR2 conductance for Zn^2+^ (81.1 ± 2.4 pS for 8 mM [Zn^2+^]_L_ vs. 127.5 ± 1.8 pS for 8 mM [Ca^2+^]_L_). The situation for luminal Mg^2+^ was slightly different because the Mg^2+^ additions resulted in a greater change in the G values (Figure 3C, left panel). This was despite that the RYR2 channel exhibited a significantly lower G for Mg^2+^ than for Ca^2+^ when testing 8 mM gradients (95.3 ± 1.4 pS vs. 127.5 ± 1.8 pS, respectively). When luminal Ca^2+^ was added, two identical cations were mixed, and therefore the G values were not changed (Figure 3C, right panel). In summary, the I_0_, G and E_rev_ results collectively indicate that the passage of Zn^2+^ through the RYR2 channel is less effective than even for Mg^2+^, despite having similar physical properties (Table 2).

### 3.3. Effects of pH When Luminal Zn^2+^ Was Added

**Figure 4:**
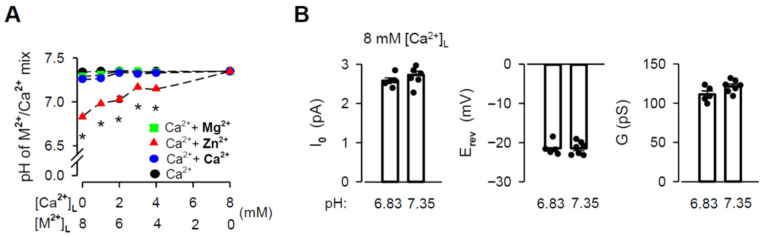
Changes in pH caused by the Zn^2+^ additions have no effects on RYR2 permeation properties. (**A**) Effects of the M^2+^ additions on pH of M^2+^/Ca^2+^ mixture (M^2+^: Mg^2+^−green square, Zn^2+^–red triangle, or Ca^2+^–blue circle). In total, 4–7 mM [M^2+^]_L_ was added to 1–4 mM [Ca^2+^]_L_, keeping the total divalent concentration constant at 8 mM. As the control, the [Ca^2+^]_L_-dependence of pH is shown (black circle). Data obtained for the pure 8 mM [M^2+^]_L_ (chloride salt) or 8 mM [Ca^2+^]_L_ (hydroxide) are also included. * Significantly different from the respective control values (one-way ANOVA with Tukey’s post hoc test). (**B**) The I_0_, E_rev_ and G values obtained for pH of 6.83 and 7.35 when 8 mM [Ca^2+^]_L_ (hydroxide) was present (unpaired Student’s *t* test). Data shown are average ± SEM of 5–7 experiments, and error bars in (**A**) are shown only when SEM is larger than symbol size. Data points shown in (**B**) are individual measurements.

The chloride salt of Zn^2+^ has been reported to change the pH of solutions because it is a weak acid [74]. Such changes could substantially affect measurements of the RYR2 G and intrinsic permeability [75,76]. To evaluate this potential source of inaccuracy, we first investigated the effects of the Zn^2+^ additions on the pH of the luminal solutions, while retaining the same volumes used for the single-channel recordings. Mg^2+^ and Ca^2+^ were examined in a similar manner. The summary data are plotted as a function of M^2+^/Ca^2+^ mixture content in Figure 4A. While Mg^2+^ and Ca^2+^ additions (4–7 mM) induced no significant changes in the pH from the control, luminal Zn^2+^ had a substantial effect with increasing its mole fraction. The pH of the luminal solution with no Ca^2+^ present (10 mM Tris, 20 mM HEPES, 50 mM KCl) dropped from 7.34 ± 0.007 to 6.83 ± 0.017 when 8 mM [Zn^2+^]_L_ was added. In contrast, there was no pH change with 8 mM [Mg^2+^]_L_ or [Ca^2+^]_l_. Importantly, experimental conditions with 8 mM [Zn^2+^]_L_ were limited to a lower pH of 6.83 because, otherwise, Zn^2+^ precipitates occurred. To test if the large decrease in pH observed for the addition of 8 mM [Zn^2+^]_L_ might produce a shift in I_0_, E_rev_ and G values, we tested 8 mM [Ca^2+^]_L_ (hydroxide) buffered to pH values of 6.83 and 7.35. Summary results presented in Figure 4B show that pH decrease was essentially without any significant effect. These data indicate that all results of mole-fraction experiments collected for luminal Zn^2+^ can be indeed interpreted as a manifestation of the intrinsic Zn^2+^ permeability displayed by the RYR2 channel.

### 3.4. Effects of Luminal Zn^2+^ on RYR2 Permeation Properties under Near-Saturating Conditions

**Figure 5:**
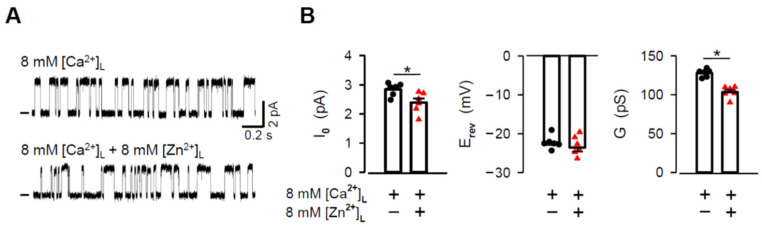
Luminal Zn^2+^ decreases the RYR2 current under near-saturating conditions. (**A**) Representative RYR2 currents, shown as upward deflections from the marked zero-current level, for 8 mM [Ca^2+^]_L_ (control, top trace) and when 8 mM [Zn^2+^]_L_ was added (bottom trace). RYR2 channels were activated by 1–2 mM caffeine in the presence of 90 nM free [Ca^2+^]_C_. Current recordings were conducted at 0 mV and are from the same channel. (**B**) The I_0_, E_rev_ and G values obtained in the absence and after luminal Zn^2+^ exposure. * Significantly different from the control values when only luminal Ca^2+^ was present (paired Student’s *t* test). Data shown are average ± SEM of 6 experiments. Data points shown in (**B**) are individual measurements (Ca^2+^–black circle and Zn^2+^/Ca^2+^–red triangle).

As seen in Figure 3C, the [Ca^2+^]_L_-dependence of the RYR2 G almost saturated at 8 mM. This indicates that all mole-fraction experiments shown in Figure 3 were performed at [Ca^2+^]_L_, which is not at the saturating portion of the G-[Ca^2+^]_L_ curve. Under such conditions, luminal Zn^2+^ would not strongly compete with Ca^2+^ for occupancy of the RYR2 pore, if at all [77,78]. To further examine the mechanisms of Zn^2+^ permeation, we extended the I_0_, E_rev_ and G measurements to include near-saturating conditions. Because 8 mM concentration was the highest possible [Zn^2+^]_L_ available for testing, we could use only a mixture with 8 mM [Ca^2+^]_L_ to allow a reasonable competition between these cations [79]. Figure 5A shows representative current traces of the same caffeine-activated RYR2 channel recorded at 0 mV in the absence and presence of luminal Zn^2+^. One can see that 8 mM [Zn^2+^]_L_ slightly reduced the current amplitude carried by 8 mM [Ca^2+^]_L_. Average results are presented in Figure 5B (left panel). While the reduction in the I_0_ was small but significant, E_rev_ changed only negligibly by ~−1.35 mV (Figure 5B, middle panel). This small shift toward more negative values was also predicted by Equation (1) for the estimated P_Ca_/P_Zn_ of 2.65 ± 0.19 (−23.97 mV theoretical vs. −23.59 ± 1.03 mV experimental). Inevitably, the G significantly decreased (Figure 5B, right panel). Of note, the aforementioned changes were not related to pH, although it dropped from 7.35 ± 0.04 to 7.058 ± 0.029 by the luminal addition of 8 mM [Zn^2+^]_L_. As shown in Figure 4B, an even larger decrease in pH from 7.35 to 6.83 did not produce any significant effect. Taken together, the decrease in I_0_ and G values by Zn^2+^ addition to almost saturating [Ca^2+^]_L_ could indeed be interpreted as a consequence of competition between two permeant cations inside the RYR2 pore, which exhibits a different intrinsic permeability and/or conductance for each cation. 

### 3.5. The Zn^2+^ Finger Located within the C-Terminus of the RYR2 Channel as a Potential Target of the Zn^2+^ Current

**Figure 6:**
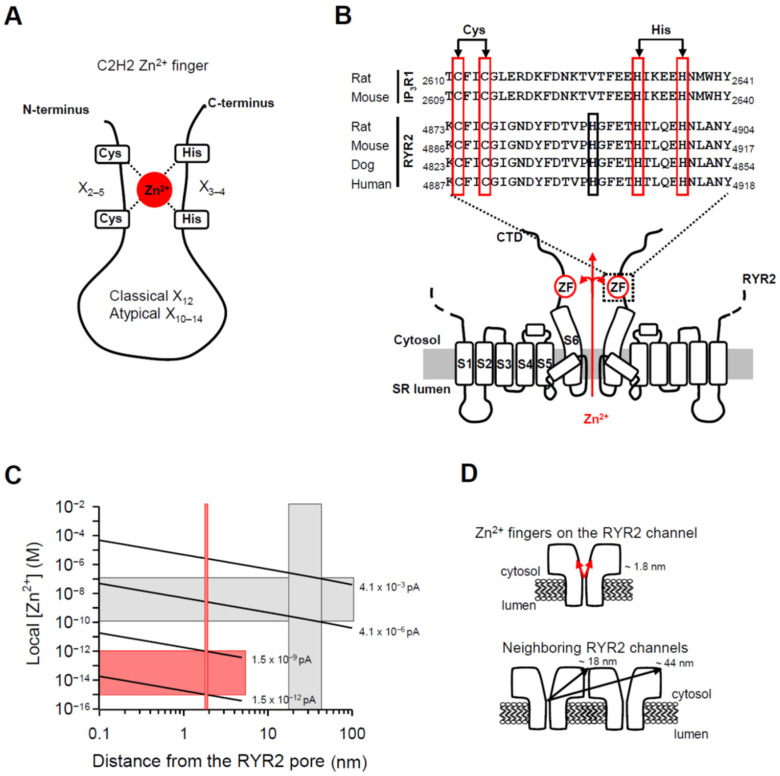
The RYR2 Zn^2+^ finger might be locally controlled by the Zn^2+^ current. (**A**) A schematic representation of the C2H2 Zn^2+^ finger consisting of two Cysteines (Cys or C) and two Histidines (His or H), which are coordinated by Zn^2+^ cation. Usually, 12 residues separate the last Cys and the first His residues (classical C2H2 pattern). In atypical C2H2 Zn^2+^ fingers which still retain the ability to bind Zn^2+^, 10–14 residues are tolerated. (**B**) Sequence alignment of the Zn^2+^ finger motifs identified within the C-terminal tail of the rat and mouse IP_3_R1 channels [81,82] and the RYR2 channel [55] from indicated mammals. The sequences were taken from the UniProtKB database. The Cys and His residues of the Zn^2+^ finger domains with uncommon spacing (16 residues separating the Cys-Cys pair from the His-His pair) are boxed in red. The conserved His in the RYR2 sequences located 11 residues from the Cys-Cys pair, but not in the IP_3_R1 sequence, is highlighted in black. The membrane topology model of two opposing RYR2 subunits with six transmembrane domains was generated from recent work on the near-atomic resolution structure of the RYR2 isoform [55]. The putative Zn^2+^-finger domains (ZF) within the C-terminal tails might be supplied with luminal Zn^2+^ emanating from the RYR2 pore in the lumen-to-cytosol direction when the channel is open. (**C**) The theoretical prediction of local Zn^2+^ in respect to a distance from the Zn^2+^ exit site (the open RYR2 pore). Zn^2+^ accumulation profile was calculated with buffer present (1 mM EGTA). The red vertical bar indicates the Zn^2+^ finger distance from a Zn^2+^ point source (~1.8 nm). The gray vertical box indicates the center-to-corner distances covering the cytosolic domain of the neighboring RYR2 channel (~18–44 nm). At a distance of ~1.8 nm, the I_0,Zn_ of 1.5 × 10^−12^–1.5 × 10^−9^ pA is required to elevate [Zn^2+^] up to the femtomolar range (horizontal red bar), sufficient to saturate the Zn^2+^ fingers. At a distance of ~18–44 nm, the I_0,Zn_ of 4.1 × 10^−6^–4.1 × 10^−3^ pA is required to reach activating [Zn^2+^] from 100 pM to 100 nM (horizontal gray bar). (**D**) Schematic depicting distances of the RYR2 Zn^2+^ fingers and cytosolic domain of the neighboring RYR2 channel from the Zn^2+^ exit site to help place the values on the X axis, shown in (**C**), in context.

In an attempt to identify a potential target of the RYR2 Zn^2+^ current, we focused on small protein domains termed the Zn^2+^ fingers whose structure is highly organized upon Zn^2+^ complexation [80]. C2H2 type with two Cysteines (Cys or C) followed by a pair of Histidines (His or H) is one of the most ubiquitous coordination sites for Zn^2+^ (Figure 6A). This configuration has been recognized within the C-terminal tail of the RYR2 channel [55] and the inositol-1,4,5-trisphosphate receptor isoform-1 (IP_3_R1) [81,82]. The IP_3_R1 channel participates in Ca^2+^ transport across the ER membrane in all cell types (reviewed in [83]). The classical C2H2 topology has the consensus sequence C-X_2–5_-C-X_12_-H-X_3–4_-H (X is for any amino acid) (reviewed in [84]). To determine and compare spacing in the C2H2 Zn^2+^ fingers of RYR2 and IP_3_R1 channels, we computed the alignment of their C-terminal sequences using the ClustalX method (Figure 6B; [85]). Instead of focusing on rat channels, we extended our analysis to other mammalians such as mouse, dog and human, which are commonly used as a source of RYR2 and IP_3_R1 channels for BLM experiments. Within this set, the first Cys and the second Cys are separated by two residues and this spacing is well conserved across mammalian RYR2 and IP_3_R1 channels. Further, the interhistidine spacing of four residues is conserved as well. Up to here, the topology of RYR2 and IP_3_R1 Zn^2+^ fingers matches the classical C2H2 pattern. However, we revealed one deviation in the distance between Cys-Cys and His-His pairs. The spacing of 16 residues is uncommon (Figure 6B). According to the consensus sequence, 12 residues are allowed, albeit, it has been shown that 10–14 residues could also be tolerated (atypical C2H2, Figure 6A). In the RYR2 sequence, specifically, we located one additional conserved His that is 11 residues away from the Cys-Cys pair and four residues from the following His. It is thus conceivable that this newly-identified His residue could act as the third Zn^2+^ ligand matching the overall spacing of the atypical C2H2 topology.

Despite a little doubt about the composition of the RYR2 Zn^2+^ finger, its location at the joint between the CTD (C-terminal domain) and the last transmembrane segment (S6) remains indisputable [55]. Since the Zn^2+^ finger domain is oriented toward the channel permeation pathway it is feasible to test the possibility that this domain is directly targeted by the Zn^2+^ current (Figure 6B). We began by calculating the free [Zn^2+^] profile around an open RYR2 pore. Considering the extremely high binding affinities of numerous Zn^2+^ fingers in the femtomolar range [86,87,88], Figure 6C shows that an extremely small Zn^2+^ current (from 1.5 × 10^−12^ pA to 1.5 × 10^−9^ pA) would be sufficient to achieve femtomolar [Zn^2+^] around the Zn^2+^ finger located ~1.8 nm from the channel pore (Figure 6D, top panel; [55]). Since the RYR2 channel has been shown to be sensitive to cytosolic Zn^2+^ [89,90] and the channel itself seems to be resistant to its own current [91], we extended our calculations also to the neighboring RYR2 channels that might be targeted via Zn^2+^ diffusion. This scenario should also receive attention because in the heart, RYR2 channels operate in small packed clusters [92,93] and at the single channel level, two or more RYR2 channels can be occasionally reconstituted, perhaps at their normal cellular spacing. Recent BLM studies have presented evidence that Ca^2+^-activated RYR2 channels are substantially modulated by cytosolic Zn^2+^ (100 pM–100 nM) [89,90]. To reach this magnitude change over the entire cytosolic domain of the neighboring channel, where Zn^2+^ binding sites must be located, the I_0,Zn_ from 4.1 × 10^−6^ pA to 4.1 × 10^−3^ pA is required (Figure 6C). Center-to-corner distances of ~18 nm and ~44 nm were taken from Liu et al. [91] and are indicated in Figure 6D (bottom panel). Such I_0,Zn_ values are 122- to 122,000-fold lower in comparison to the physiologically relevant amplitude of the Ca^2+^ current (~0.5 pA) flowing through the RYR2 channel [77,78]. Thus, our calculations suggest that RYR2 function might be significantly affected by almost negligible Zn^2+^ current of more than or equal to 4.1 × 10^−6^ pA, which could be reachable in cardiomyocytes.

### 3.6. Displacement of Zn^2+^ from the RYR2 Zn^2+^ Finger

**Figure 7:**
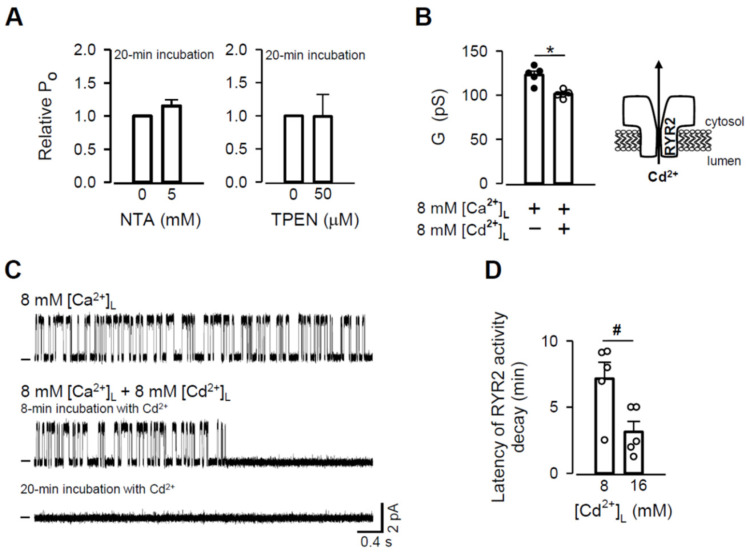
Zn^2+^ displacement from the RYR2 Zn^2+^ finger. (**A**) Effects of NTA (left panel) and TPEN (right panel) added to the cytosolic solution on RYR2 P_O_ as a consequence of a decrease in [Zn^2+^]_C_ (paired Student’s *t* test). (**B**) The effect of Cd^2+^ addition (8 mM) on the RYR2 G under near-saturating conditions when 8 mM [Ca^2+^]_L_ was present. * Significantly different from the control values when only luminal Ca^2+^ was present (paired Student’s *t* test). (**C**) Representative RYR2 currents, shown as upward deflections from the marked zero-current level, for 8 mM [Ca^2+^]_L_ (control, top trace) and when 8 mM [Cd^2+^]_L_ was added (two bottom traces). RYR2 channels were activated by 1–2 mM caffeine in the presence of 90 nM free [Ca^2+^]_C_. Current recordings were conducted at 0 mV and are from the same channel. (**D**) Latency of RYR2 activity decay as a function of [Cd^2+^]_L_. ^#^ Significantly different from 8 mM [Cd^2+^]_L_ (unpaired Student’s *t* test). Data shown are average ± SEM of 5–7 experiments. Data points shown in (**B**) and (**D**) are individual measurements (Ca^2+^–black circle and Cd^2+^/Ca^2+^–white circle).

To further test the idea that the Zn^2+^ current supplies Zn^2+^ cations essential for the stabilization of the RYR2 Zn^2+^ finger, we assessed the accessibility of this Zn^2+^ binding site from either channel side (cytosolic and luminal) by displaying Zn^2+^ from its structure. As a nondestructive readout for this event, we monitored RYR2 P_O_ because it has been evidenced that the Zn^2+^ finger is fundamental to the channel activation by caffeine [55]. First, we tested whether Zn^2+^ chelating agents such as NTA or TPEN could compete for Zn^2+^ when applied to the RYR2 cytosolic side. Based on the purity assays of ultrapure chemicals (Sigma-Aldrich) and the strong binding affinity of EGTA to Zn^2+^, we estimated that ~2.13 pM [Zn^2+^]_C_ should have always been present in the cytosolic solution. When 5 mM NTA was added, [Zn^2+^]_C_ dropped down to 0.5 pM. During 20-min incubation, the RYR2 P_O_ did not change significantly (Figure 7A, left panel). Although, 5 µM TPEN decreased free [Zn^2+^]_C_ to the attomolar range (7.2 aM = 0.0072 fM), no significant change in the channel activity was observed during 20-min exposure (Figure 7A, right panel). Insufficiently high Zn^2+^ affinities of NTA and TPEN appear to be unlikely because binding affinities of numerous Zn^2+^ fingers are in the femtomolar range [86,87,88]. Presumably, a substantially longer incubation (it could reach hours) was needed to remove obviously tightly bound Zn^2+^ in the RYR2 Zn^2+^ finger, which could also be less approachable sitting deep in the RYR2 cytosolic vestibule. A short BLM lifetime, however, limited such demanding conditions. In a further attempt to displace Zn^2+^, we assessed whether a well-known competition between Zn^2+^ and Cd^2+^ in different eukaryotic Zn^2+^ fingers [94,95,96,97] could be a more direct approach. Given the comparable physical properties of Cd^2+^ with those of permeant Ca^2+^ and Mg^2+^ (Table 2), Cd^2+^ passage through the RYR2 channel might be expected. Indeed, we found that the luminal addition of 8 mM [Cd^2+^]_L_ to 8 mM [Ca^2+^]_L_ significantly decreased the RYR2 G, thus reflecting Cd^2+^ permeation (Figure 7B), as we evidenced for Zn^2+^ under the same conditions (Figure 5). This finding was crucial because it allowed us to approach the Zn^2+^ finger from the RYR2 luminal side by the Cd^2+^ current in the lumen-to-cytosol direction. Importantly, a drop in pH from 7.350 ± 0.010 to 7.234 ± 0.016 when 8 mM [Cd^2+^]_L_ was added did not contribute to a decrease in G because a much larger pH change from 7.35 to 6.83 was essentially without effect (Figure 4B). In Figure 7C (top trace), the Ca^2+^ current was only passing the caffeine-activated RYR2 channel in the presence of 8 mM [Ca^2+^]_L_. After the addition of 8 mM [Cd^2+^]_L_, a sudden decay of channel activity occurred after 8-min incubation and persisted within 20 min of exposure (Figure 7C, bottom two traces). Figure 7D shows that the latency of RYR2 activity decay varied considerably with [Cd^2+^]_L_. As [Cd^2+^]_L_ increased from 8 mM to 16 mM, the channel activity was suppressed within a shorter time due to a greater ability of Cd^2+^ to substitute for Zn^2+^. Importantly, RYR2 activity was not recovered when caffeine concentration was substantially increased to 6–7 mM. This indicates that RYR2 sensitivity to caffeine was not attenuated as a result of Cd^2+^/Ca^2+^ competition directly at the RYR2 luminal side, as previously shown for Sr^2+^, Mg^2+^, or Ba^2+^ (when competing with 1 mM luminal Ca^2+^) [51]. Rather, the Cd^2+^ current destabilized the Zn^2+^ finger by replacing Zn^2+^ and thus promoting the decay of channel activity.

## 4. Discussion

There has been a rapidly growing interest in the field of Zn^2+^ biology in the last ten years because of significant advances in the knowledge of Zn^2+^ chemistry and biochemistry. Moreover, defective Zn^2+^ cellular homeostasis has been implicated in the pathophysiology of cancer and diabetes, common diseases which greatly affect global health. This has further stimulated an intensive research effort to understand the importance of Zn^2+^ in a diverse spectrum of biological functions. In mammalian cells, large amounts of Zn^2+^ accumulate in various cellular compartments and organelles [34,35,36], mainly as a structural or catalytic factor directly regulating a great number of protein functions. To be candidates for Zn^2+^ signaling, intracellular Zn^2+^ storages must have proteins for both Zn^2+^ release and reuptake. Although, Zn^2+^ transporters such as ZIP and ZnT with subcellular distribution have been identified in various mammalian cells (reviewed in [17,18,19]), a limited number of studies have been conducted in cardiomyocytes. However, at least one member of the ZIP family responsible for Zn^2+^ release to the cytoplasm and one member of the ZnT family mediating Zn^2+^ transport in the opposite direction have been found [27,40] and localized to the cardiac SR membrane [41]. Although, these transporters assumingly participated in Zn^2+^ signaling originating from the SR [8,38], we put forward the possibility that the cardiac ryanodine receptor (RYR2) located in the SR membrane also contributes. It has been known for many years that the RYR2 channel is responsible for a massive release of Ca^2+^ from the SR required for muscle contraction (reviewed in [98]). Its role in Zn^2+^ signaling has however been overlooked. Tuncay et al. [8] revealed a significant suppression of Zn^2+^ transients by ryanodine in electrically stimulated cardiomyocytes. Ryanodine is a plant alkaloid that binds to all three isoforms of the RYR channel with high affinity and either holds the channel open in a state of reduced conductance or causes a complete inhibition, depending on its concentration [59,99,100,101]. According to Tuncay et al. [8], Zn^2+^ transients mostly resulted from an increase in [Ca^2+^]_C_ and subsequent Zn^2+^ displacement from intracellular binding sites. In such a scenario, Zn^2+^ transients would be suppressed in the presence of ryanodine, because Ca^2+^ release from the SR mediated by the RYR2 channel would be compromised. However, considering similar physical properties of Zn^2+^ with those of permeant Mg^2+^ (Table 2), the Zn^2+^ release through the RYR2 channel which would inevitably accompany Ca^2+^ release, could be an alternative explanation. Our study was therefore designed to investigate whether Zn^2+^ permeates the RYR2 channel reconstituted into the BLM. 

### 4.1. Permeation Properties of the RYR2 Channel for Zn^2+^

In this study, the permeation properties of the rat RYR2 channel were examined under various ionic conditions. As shown in Figure 1, the Zn^2+^ currents through the RYR2 channel in the luminal-to-cytosol direction were recorded for the first time. Our results are compatible with several studies reporting a weak Zn^2+^ conductance for voltage-gated Ca^2+^ channels [102,103,104,105]. When 8 mM [Zn^2+^]_L_ was present and used as a sole charge carrier, the RYR2 I_0,Zn_ was 0.927 ± 0.093 pA. It is comparable to, but significantly smaller than, those obtained for luminal Ca^2+^ (2.835 ± 0.094 pA) or even Mg^2+^ (1.822 ± 0.079 pA) under similar conditions. Accordingly, the G values fall in the sequence Ca^2+^ (127.5 ± 1.8 pS) > Mg^2+^ (95.3 ± 1.4 pS) > Zn^2+^ (81.1 ± 2.4 pS). Such differences between Zn^2+^ and Mg^2+^ were unexpected as these cations have similar physical properties relevant to ion channel permeation (Table 2). To gain a better understanding, we evaluated the Zn^2+^ permeability coefficient relative to Ca^2+^. We performed mole-fraction experiments with a mixture of Zn^2+^ and Ca^2+^ on the luminal side (8 mM total concentration) and monitored the changes in RYR2 permeation properties. Zn^2+^ additions to the luminal solution resulted in specific changes when we used non-saturating concentrations of luminal Ca^2+^ in respect to the RYR2 conductance (<8 mM). The I_0_ and G were increased while the E_rev_ was shifted towards more negative values (Figure 3). The changes were more pronounced in mixtures with the highest mole fractions of Zn^2+^. Under near-saturating conditions, where a competition between Zn^2+^ and Ca^2+^ could occur within the RYR2 permeation pathway [79], the I_0_ and G were significantly decreased, but no change was revealed for E_rev_ when 8 mM [Zn^2+^]_L_ was added to 8 mM [Ca^2+^]_L_ (Figure 5). Since the I_0_ and G did not drop below the values obtained for pure luminal Zn^2+^, this phenomenon cannot be interpreted as the anomalous mole fraction effect occurring when the values of I_0_ or G are lower in a mixture of two ions than in the pure solutions of individual ions at the same total concentration. To make such conclusion, we assumed that the permeation properties of the RYR2 channel at 8 mM and 16 mM [Zn^2+^]_L_ (the latter is not reachable at pH ~7.00) are similar. This was not an unreasonable proposal, as 8 mM concentration of divalent cations was found to be near-saturating. 

In comparison to permeant Mg^2+^, Zn^2+^ displayed much less ability to contribute to permeation properties. The permeability coefficient for Zn^2+^ (P_Ca_/P_Zn_ = 2.65 ± 0.19), estimated by fitting dependence of E_rev_ on a composition of Zn^2+^/Ca^2+^ mixture by Equation (1), was found to be ~2.3-fold lower than that of Mg^2+^ (P_Ca_/P_Mg_ = 1.146 ± 0.071). Because the permeability coefficient has been shown to be a diffusion component of channel selectivity and reflects how well particular ions pass through the channel pore [106], our results clearly indicate that the RYR2 channel can differentiate, albeit only moderately, between Zn^2+^ and Mg^2+^ or Ca^2+^, providing a slower path to Zn^2+^. In respect to G, it has been proven that this parameter contains information not only about ion movement throughout the channel pore, but it also measures how well particular ions enter and exit the channel [62,106]. Since Zn^2+^ and Mg^2+^ exhibit a similar hydration enthalpy (Table 2), critically implicated in ion entry into the narrowest region of the channel pore [52,62], and as Zn^2+^ moves through the RYR2 channel more slowly than Mg^2+^, a lower G_Zn_ is an inevitable consequence.

### 4.2. Physiological Implications

**Figure 8:**
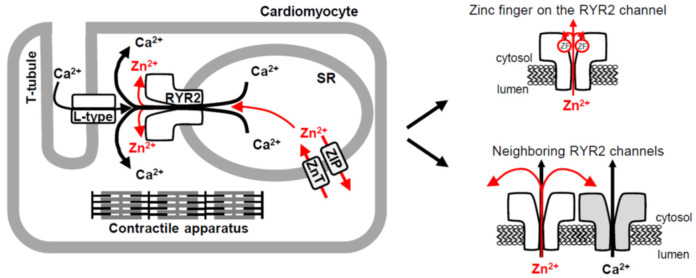
The RYR2 channel provides a novel pathway for rapid Zn^2+^ transport in cardiomyocytes. Schematic illustrating three pathways (one novel) for Zn^2+^ transport across the cardiac SR membrane. Two Zn^2+^ transporter protein families, ZIP and ZnT, play an essential role in cardiac Zn^2+^ homeostasis and mediate a slow-rate transport. In the heart, Zn^2+^ release through the RYR2 channel, accompanying a robust Ca^2+^ release triggered by small Ca^2+^ entry through the L-type Ca^2+^ channel, could also contribute to global and/or local Zn^2+^ signaling which requires a fast-rate transport. At the local scale, the Zn^2+^ current flowing through the RYR2 channel might target its intrinsic Zn^2+^ finger situated within the C-terminal tail of all four RYR2 subunits. In addition, the RYR2 channel (colored gray) might be stimulated by Zn^2+^ cations emanating from the neighboring RYR2 channel (colored white). Arrows indicate the direction of Zn^2+^ (red) and Ca^2+^ (black) mobilization.

Ion channels are predominantly involved in rapid signal transduction because ion movement through ion channels is usually much faster than passive transport down the electrochemical gradient mediated by carrier-type transporters [107]. During each heartbeat, RYR2 channels are responsible for the release of a huge amount of Ca^2+^ from the SR lumen. Our study shows that the same route is also suitable for Zn^2+^ mobilization from the SR, and thus Zn^2+^ might contribute to the RYR2 current of ~0.5 pA [77,78] during cardiac excitation-contraction coupling, albeit primarily driven by a 10,000-fold Ca^2+^ gradient across the SR membrane (100 nM in the cytosol, 1 mM in the SR lumen [108,109]). This seems reasonable, if we consider the extremely low free Zn^2+^ level of ~100 pM in the cytosol of cardiomyocytes [3,110] and more than 60-fold higher free [Zn^2+^] in the cardiac SR lumen (>6 nM, [110]). The existence of a Zn^2+^ gradient, albeit ~166-fold smaller than for Ca^2+^, together with a considerable RYR2 permeability for Zn^2+^, is compatible with the biological significance of the Zn^2+^ current through the RYR2 channel. At the cellular level, Tuncay et al. [8] support this idea by visualizing ryanodine-sensitive Zn^2+^ transients in cardiomyocytes having similar kinetics to those of Ca^2+^. Furthermore, both Ca^2+^ and Zn^2+^ transients required a small Ca^2+^ influx from the extracellular space through the L-type Ca^2+^ channel in the plasma membrane. Thus, it seems reasonable to propose that the RYR2 channel has a real Zn^2+^ transport function in cardiomyocytes (Figure 8), but the cellular target for this Zn^2+^ release has not been established. Certainly, the release does not trigger contraction because Zn^2+^ only negligibly interacts with proteins of the contractile machinery [111]. However, 100 pM–100 nM Zn^2+^ has been shown to shape Ca^2+^ release by amplifying the Ca^2+^-induced RYR2 activity [89,90]. According to our simple calculations, the Zn^2+^ current of more than or equal to 4.1 × 10^−6^ pA (~0.0008% of the RYR2 current in cardiomyocytes) should be sufficient for the building up of activating [Zn^2+^] near neighboring RYR2 channels (Figure 6C). This estimation suggests that almost negligible Zn^2+^ current mediated by one RYR2 channel could amplify Ca^2+^ release through neighboring RYR2 channels. This regulatory process might be impaired in cardiovascular diseases, which are often associated with Zn^2+^ deficiency [39,112,113,114]. It may arise from low intake, malabsorptive syndromes, or the administration of various medications (reviewed in [115]). In Zn^2+^-deficient cardiomyocytes, intracellular Zn^2+^ stores including the SR have been indeed found to be substantially depleted of Zn^2+^ [116]. One would then expect that the Zn^2+^ gradient across the SR membrane will be reduced, resulting in a compromised Zn^2+^ release. This implies that appropriate Zn^2+^ supplementation might be beneficial in the management of cardiovascular diseases, directly affecting Ca^2+^ signaling fundamental to the cardiac contraction. So far, a few studies have reported an improvement in symptoms of heart failure with Zn^2+^ supplementation [116,117,118]. Some clues on the physiological relevance of the RYR2 Zn^2+^ current, can also be found in work of Atar et al. [104], where changes in free [Zn^2+^]_C_ have been linked to the activation of transcription in electrically stimulated cardiomyocytes, given the catalytic and structural roles of Zn^2+^ in DNA- and RNA-binding proteins.

Numerous proteins with a variety of functions, including transcription, protein degradation, and DNA repair harbor small structured domains, the Zn^2+^ fingers, whose stability is dramatically improved by Zn^2+^ binding. The C2H2 Zn^2+^ finger with two Cys (C) followed by a pair of His residues (H) (Figure 6A) has also been identified within the C-terminal tail of structurally related IP_3_R1 [81,82] and RYR2 channels [55] (Figure 6B). When individual or combined Cys and His residues were mutated, IP_3_R1 function was completely abolished highlighting the critical role of the C-terminus in channel gating [81,82]. The C-terminal region is highly conserved in both the IP_3_R and RYR families [119] and therefore it is not surprising that similar results were obtained for the RYR2 channel. The channel completely lost its ability to gate when both Cys residues and only the first of a His-His pair were individually substituted [55]. Surprisingly, mutation of the final His residue caused no change and the RYR2 channel retained its function. This is in sharp contrast to the IP_3_R1 channel, where a critical importance of the final His residue in the Zn^2+^ finger sequence has been reported [82], albeit the spacing between the Cys-Cys and His-His pairs is uncommon (16 instead of classical 12 or atypical 10–14 residues, Figure 6B). The same deviation has also been demonstrated for the RYR2 channel [55], although we found one nearer conserved His, 11 residues away from the Cys-Cys pair (atypical C2H2 pattern), that could potentially serve as a third Zn^2+^ ligand when pairing with the following His (Figure 6B). To address such uncertainty in the composition of RYR2 Zn^2+^ finger, the mutation of the newly-identified His residue should be studied in the context of RYR2 function and Zn^2+^ binding properties.

The Zn^2+^ finger domain of each of the four RYR2 subunits impinges toward the central vertical axis of the channel permeation pathway, thus it may be well supplied with the Zn^2+^ current to ensure a proper channel function (Figure 8). In agreement with this prediction, the Cd^2+^ current passing the RYR2 channel in the lumen-to-cytosol direction caused a sudden decay of RYR2 activity. Since this action was specific for Cd^2+^ because the luminal additions of other divalent cations never had such detrimental effects [50,51], it was highly likely due to Zn^2+^ to Cd^2+^ replacement in the RYR2 Zn^2+^ finger. Obviously, Zn^2+^ displacement by Cd^2+^ was not structurally tolerated, and therefore RYR2 function was abolished. If the RYR2 Zn^2+^ current was fundamental to stabilization of the Zn^2+^ finger structure, one could ask why the channel function was not impaired in the absence of luminal Zn^2+^ when only Ca^2+^ or Mg^2+^ currents were carried by the channel. The answer could be found in Zn^2+^ impurities coming from otherwise ultrapure chemicals. We estimated that ~2.13 pM and ~0.8–1.0 nM free [Zn^2+^] was present in the cytosolic and luminal solutions, respectively. In generally, eukaryotic Zn^2+^ fingers possess the extremely high binding affinities in the femtomolar range [86,87,88], therefore, picomolar [Zn^2+^] on the RYR2 cytosolic side should have been sufficient to saturate the Zn^2+^ finger, albeit the deep position of this Zn^2+^-stabilized domain within the cytosolic portion of the RYR2 permeation pathway could considerably impair this process. We tested this idea by adding strong Zn^2+^ chelators such as NTA and TPEN to reach the atto-femtomolar free [Zn^2+^]_C_, and thus facilitate Zn^2+^ displacement from the channel. This strategy was, however, not successful. One reason was probably the specific binding properties of the RYR2 Zn^2+^ finger. It is reasonable to assume that Zn^2+^ has to be tightly bound even under conditions of a transient depletion in Zn^2+^ because the RYR2 channel plays a dominant role in cardiac excitation-contraction coupling [42,43,44], and thus a proper RYR2 function is of particular importance. On the other hand, the ~470-fold Zn^2+^ gradient across the BLM, which was always present due to Zn^2+^ impurities, also likely contributed. Our calculations suggest that almost negligible Zn^2+^ current (≥1.5 × 10^−12^ pA) is required to accumulate femtomolar [Zn^2+^] near the Zn^2+^ finger position (~1.8 nm from the pore). In cardiomyocytes, this extremely small current is seemingly also reachable, albeit Zn^2+^ movement will be driven by a smaller Zn^2+^ gradient compared to BLM experiments. Taken together, it appears that the RYR2 Zn^2+^ finger is more accessible from the luminal than the cytosolic side of the channel since Cd^2+^ current (from lumen to cytosol) replaced Zn^2+^ bound in the Zn^2+^ finger domain even in the presence of saturating [Zn^2+^]_C_ in the cytosolic solution. In contrast, a substantial decrease in [Zn^2+^]_C_ to the non-saturating range had no effect on Zn^2+^-RYR2 interactions when a subtle Zn^2+^ current in the lumen-to-cytosol direction occurred.

## 5. Conclusions

Here, we identify the RYR2 channel as a novel Zn^2+^ transporting protein in the SR membrane that might play a role in local and/or global Zn^2+^ signaling in cardiomyocytes, considering much faster ion movement through ion channels than the passive transport mediated by carrier-type transporters. The results demonstrate that the RYR2 channel itself could be regulated by its own Zn^2+^ current. Since Zn^2+^-binding domains, such as the Zn^2+^ fingers, often display extremely high binding affinities in the femtomolar range a subtle Zn^2+^ current in the lumen-to-cytosol direction was predicted to be sufficient for the saturation of the Zn^2+^ finger situated within the C-terminal tail of each of the four RYR2 subunits. It is not an unreasonable proposal, because the RYR2 Zn^2+^ finger is directed toward the permeation pathway, and thus it will inevitably be targeted by the Zn^2+^ current.

## Figures and Tables

**Table 1:** Effects of luminal M^2+^ (M^2+^: Mg^2+^, Zn^2+^ or Ca^2+^) on gating parameters of the RYR2 channel at P_O_ ~ 0.5.

Luminal M^2+^	P_O_	F (Hz)	T_O_ (ms)	T_C_ (ms)
8 mM [Ca^2+^]_L_	0.4947 ± 0.0059	10.29 ± 1.04 ^#^	49.03 ± 4.73 ^#^	49.81 ± 7.04 ^#^
8 mM [Mg^2+^]_L_	0.5137 ± 0.0040	38.4 ± 2.4 *	13.54 ± 0.77 *	12.94 ± 0.99 *
8 mM [Zn^2+^]_L_	0.526 ± 0.010	36.99 ± 1.03 *	14.74 ± 0.64 *	12.17 ± 0.51 *
1 mM [Ca^2+^]_L_ + 7 mM [Zn^2+^]_L_	0.495 ± 0.015	11.43 ± 0.66 ^#^	41.8 ± 1.7 ^#^	45.22 ± 4.37 ^#^

Data are presented as average ± SEM of 5 different experiments. * Significantly different from 8 mM [Ca^2+^]_L_; **^#^** significantly different from 8 mM [Zn^2+^]_L_ (one-way ANOVA with Tukey′s post hoc test).

**Table 2:** Physical characteristics of divalent cations implicated in permeation through ion channels.

Divalent Cation	Ionic Radius ^a^ (pm)	Charge Density ^b^ (C mm^−3^)	Enthalpy of Hydration ^c^ (kJ mol^−1^)
Ca^2+^	99	79	−1577
Mg^2+^	65	278	−1921
Zn^2+^	71	214	−2046
Cd^2+^	91	102	−1807

^a^ Ionic radius taken from [72]. ^b^ Charge density was calculated according to the formula 2e/(4/3)πr^3^, where r is the ionic radius and e is 1.602 × 10^−19^ C. ^c^ Enthalpy of hydration taken from [73].

## Data Availability

The datasets generated for this study are available on request to the corresponding authors.
